# Game-based learning to improve diagnostic accuracy: a pilot randomized-controlled trial

**DOI:** 10.1515/dx-2023-0133

**Published:** 2024-01-30

**Authors:** Daniel J. Morgan, Laura Scherer, Lisa Pineles, Jon Baghdadi, Larry Magder, Kerri Thom, Christina Koch, Nick Wilkins, Mike LeGrand, Deborah Stevens, Renee Walker, Beth Shirrell, Anthony D. Harris, Deborah Korenstein

**Affiliations:** Department of Epidemiology and Public Health, University of Maryland School of Medicine, Baltimore, MD, USA; VA Maryland Healthcare System, Baltimore, MD, USA; Adult and Child Consortium of Health Outcomes Research and Delivery Science (ACCORDS), University of Colorado School of Medicine, Aurora, CO, USA; Division of Cardiology, University of Colorado School of Medicine, Aurora, CO, USA; Center of Innovation for Veteran-Centered and Value-Driven Care, VA Denver, Denver, CO, USA; Division of General Internal Medicine, University of Maryland School of Medicine, Baltimore, MD, USA; Code in the Schools, Baltimore, MD, USA; Firaxis Games, Baltimore, MD, USA; Visual Communication Design, Thomas Jefferson University, Philadelphia, PA, USA; Department of Medicine, Icahn School of Medicine at Mount Sinai, New York, NY, USA

**Keywords:** medical diagnosis, medical education, online game-based learning

## Abstract

**Objectives:**

Perform a pilot study of online game-based learning (GBL) using natural frequencies and feedback to teach diagnostic reasoning.

**Methods:**

We conducted a multicenter randomized-controlled trial of computer-based training. We enrolled medical students, residents, practicing physicians and nurse practitioners. The intervention was a 45 min online GBL training vs. control education with a primary outcome of score on a scale of diagnostic accuracy (composed of 10 realistic case vignettes, requesting estimates of probability of disease after a test result, 0–100 points total).

**Results:**

Of 90 participants there were 30 students, 30 residents and 30 practicing clinicians. Of these 62 % (56/90) were female and 52 % (47/90) were white. Sixty were randomized to GBL intervention and 30 to control. The primary outcome of diagnostic accuracy immediately after training was better in GBL (mean accuracy score 59.4) vs. control (37.6), p=0.0005. The GBL group was then split evenly (30, 30) into no further intervention or weekly emails with case studies. Both GBL groups performed better than control at one-month and some continued effect at three-month follow up. Scores at one-month GBL (59.2) GBL plus emails (54.2) vs. control (33.9), p=0.024; three-months GBL (56.2), GBL plus emails (42.9) vs. control (35.1), p=0.076. Most participants would recommend GBL to colleagues (73 %), believed it was enjoyable (92 %) and believed it improves test interpretation (95 %).

**Conclusions:**

In this pilot study, a single session with GBL nearly doubled score on a scale of diagnostic accuracy in medical trainees and practicing clinicians. The impact of GBL persisted after three months.

## Introduction

Diagnostic error will impact most people during their lifetime [1]. Inappropriate understanding of probability of disease given a positive or negative test result can lead to diagnostic errors and thus is a critical barrier to progress [2], [3], [4]. Diagnostic medical-decision making, such as this, is a form of judgement under uncertainty, which is prone to biases if not objective [5], [6], [7]. Diagnostic decision making and test interpretation is predominantly taught as a mathematical calculation with formulas or 2×2 tables [8, 9]. Methods for teaching diagnostic probability that have shown promise include using decision analysis tree natural frequencies with or without graphics to make probability more intuitive [10], [11], [12]. Better diagnostic education has been called for but tools using these advanced approaches are lacking [8, 9].

Educational games use repetitive, rapid decision-making with immediate feedback to train skills [13]. They have been widely used to improve skills in chess and gambling, and medicine, where applications included simulations in emergency settings [14]. These games are more efficient than problem-based learning and may be superior for training intuitive associations [15]. Games have targeted heuristics to change thinking processes inherent in clinical medicine, suggesting broad future application [14, 16].

We report a pilot study evaluating an online game designed to improve diagnostic testing skills in medical students, residents and practicing clinicians. The intervention consisted of watching a short video and playing a game teaching estimates of probability of disease before and after testing. The game sought to train clinician intuitive gestalt without formal calculations. A control intervention consisted of traditional education materials. We evaluated the effect immediately after training, at one- and three-month follow-up.

## Methods

### Participants

We enrolled medical students, internal medicine residents and practicing clinicians at two hospitals. Potential participants were contacted through a group email to medical students, another email to internal medicine residents and emails and direct recruitment of practicing clinicians (physicians and nurse practitioners (NP)).

**Figure 1:**
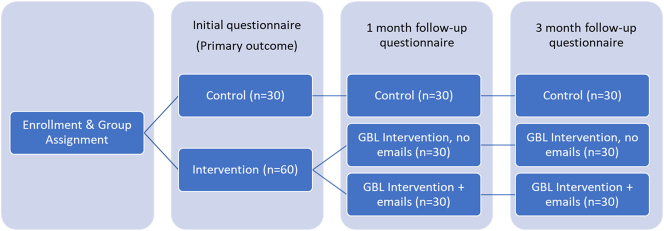
Overview of participant assignment and frequency of assessments during the study.

Participants were randomized to intervention (GBL; 30 participants), intervention with cases to remind participants of game-based learning (30 participants) or control (30 participants). In total, participants were assigned to three groups in a 1:1:1 fashion. These groups were GBL intervention, GBL intervention plus emails, and control (see Figure 1, study overview). Randomization was stratified by type of participant (student, resident, clinician). After enrollment, participants were given a link to a Qualtrics survey that contained links to individual interventions and follow up questionnaires. We provided gift cards at three points of engagement: a $100 gift card after completing the initial assessment and $50 gift cards after the one- and three-month post-intervention assessments.

### Intervention

The game was developed iteratively with clinician feedback and is publicly available (https://bird.testingwisely.com/). It requires users to estimate probability of diagnosis based on 10 simplified case presentations. Playing one game takes 5 min or less. Immediate feedback on each question is delivered using natural frequency methods with a score, tips, and comparisons to other users. The game has a short tutorial and different play options.

The entire GBL intervention instructed participants to (1) visit the testingwisely.com website and review materials, (2) watch a 4 min video explaining natural frequencies to determine post-test probability (Episode 4, https://www.testingwisely.com/educational-videos); (3) play the game tutorial and two games. The initial GBL intervention was expected to take 45 min or less. Participants attested to completing each step of the intervention.

After assessing the primary outcome, half of the GBL intervention group was randomized to receive weekly emails containing a clinical case emphasizing points from GBL.

### Control exposure

The control condition required a similar amount of time, and standard materials taught diagnosis using traditional calculations and 2×2 table methods. Materials were provided in the control Qualtrics survey and included reading the UpToDate online medical textbook testing chapter [17] and two highly-viewed YouTube videos on diagnostic testing (https://youtu.be/Z5TtopYX1Gc and https://youtu.be/dHj7ygeqelw). Participants attested to completing each step of the control.

### Measures

#### Primary outcome

A diagnostic accuracy score based on participant responses to 10 case vignettes. Each vignette asked participants to estimate the post-test probability of disease based on results of real tests and diagnoses (see Supplemental Material, Appendix 1). Responses were multiple choice; 10 points were awarded if correct and four points for the closest to the correct answer among the incorrect options. The total possible score was 100 points.

#### Secondary outcomes

Comparisons of diagnostic accuracy score at one-month and three-month follow-up.

In addition to a diagnostic accuracy score, participants provided demographic information and open-ended feedback.

### Statistical methods, analyses and expected outcomes

The primary outcome compared the continuous accuracy score in GBL intervention vs. control subjects. The distribution of this score was compared between the GBL intervention or control at the initial assessment, and all three groups at the one-month and three-month assessments. Point estimates and p-values were based on a longitudinal regression model fit by maximum likelihood.

### Sample size considerations

This was a pilot study with the goal of making estimates around effect size, optimal intervention and feasibility of enrolling different groups.

This study was deemed exempt by the Institutional Review Board (IRB) of note. Because this was a pilot study, it was not registered in clinicaltrials.gov consistent with guidance for pilot studies [18].

## Results

### Demographics

Among the 90 participants, 30 were medical students, 30 residents and 30 practicing clinicians. Demographics are provided in Table 1.

**Table 1:** Demographic information on the 90 participants participating in the study.

	Control (30)	GBL intervention (60)
Female gender	17 (56.7 %)	39 (65.0 %)
Race		
Asian	8 (26.7 %)	27 (45 %)
Black	3 (10 %)	4 (6.7 %)
White	17 (56.7 %)	30 (50.0 %)
Other race	3 (10 %)	3 (5.0 %)
Hispanic or Latino	3 (10 %)	3 (5.0 %)
Practitioner type		
Medical student	10 (33.3 %)	20 (33.3 %)
Resident	10 (33.3 %)	20 (33.3 %)
Practicing clinician	10 (33.3 %)	20 (33.3 %)
MD/DO	10 (33.3 %)	17 (28.3 %)
NP	0	3 (5.0 %)
Report current use of online resources	28 (93.3 %)	54 (90.0 %)

NP, nurse practitioners.

### Follow-up

All participants completed the initial evaluation. At one month, 71/90 completed follow-up (79 %) and at three months, 66/90 completed follow-up (73 %). Notably, follow-up was worse in those receiving intervention plus emails than intervention alone (19/30 vs. 24/30), likely as a participant noted, because of routinely ignoring study emails in the group that received additional emails.

### Primary outcome

**Figure 2:**
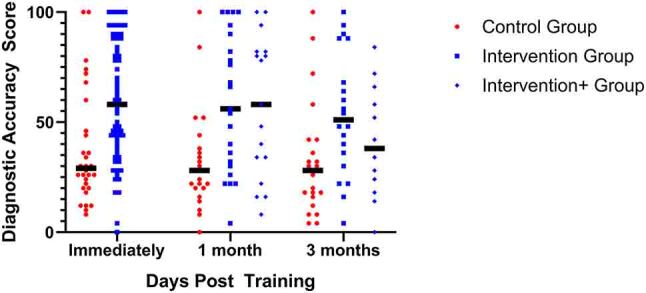
Impact of game-based learning with or without follow-up emails (intervention) vs. a standard control education immediately after game-based learning (primary outcome) and at one- and three-month follow-up. Horizontal bars represent group means.

The game-based learning intervention improved mean diagnostic accuracy; mean scores were 59.4 (of 100) in those who underwent game-based learning vs. 37.6 in controls. The estimated initial difference in score based on the longitudinal regression model was found to be 21.8 points (95 % confidence interval 9.8 to 33.9 points, p=0.0005). This is based on a longitudinal regression model accounting for the correlation between repeated measures on the same participant (see Figure 2).

### Secondary outcomes

At one-month and three-month follow up, there were three groups: GBL intervention, GBL intervention plus emails and control. At one-month follow up, GBL (mean score 59.2) and GBL plus emails (mean score 54.2) performed better than control (33.9), p=0.024. There was no difference between GBL with or without emails (p=0.99). At three-month follow up, game-based learning (mean score 56.2) and GBL plus emails (mean score 42.9) were not significantly different than control (35.1), p=0.077. There was no significant difference between GBL with and GBL without emails (p=0.091) (see Figure 2).

Medical students statistically performed no differently than residents or clinicians in practice, although the effect of GBL appeared potentially less in practicing clinicians than other groups. Initial performance after GBL intervention (mean 68.0 residents, vs. 62.1 students, vs. 48.2 practicing clinicians) (p=0.067).

### Experience of game-based learning

**Figure 3:**
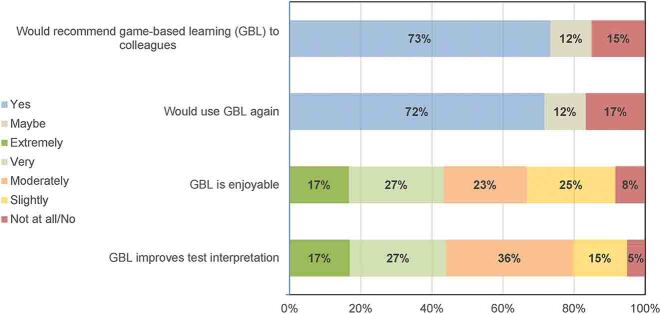
Participants perceptions of game-based learning (GBL). Recommending or using GBL could be answered yes, maybe or no. Opinion of enjoyability and improving test interpretation could be extremely, very, moderately, slightly, not at all/no.

Participants were asked if they would recommend GBL or use GBL again (possible answers yes/maybe/no) and their degree of support for statements about GBL (possible answers not at all/slightly/moderately/very/extremely). Participants were generally positive about GBL being enjoyable and improving test interpretation and most would recommend or use again (see Figure 3).

### Qualitative responses to game-based learning

Intervention group participants were asked to give optional feedback on GBL. All participants who completed the intervention provided feedback (60/60). Themes and examples are described in Table 2.

**Table 2:** Responses of participants randomized to GBL interventions to optional, open-ended questions, organized by most common themes.

Question (# responses)	Theme	Example
Describe the best part of GBL (58/60 GBL intervention participants responding)	GBL improves number sense for probabilities	“The game really helped me develop a quick method to evaluate the meaning of a test given a pre-test probability, sensitivity, specificity, and test result”
	GBL is easy and intuitive to use	“Very intuitive interface that was easy to navigate. Feedback was helpful to gauge patterns with right/wrong answers”
	GBL would help with board exams	“I wish it was something I looked at when studying for step 3”
What was the worst part of or what you would change about GBL? (53/60)	The game was hard	“It was frustrating when I got the wrong answers, but I started to pick up on patterns as I continued to play,”
	A timer was challenging	“Trying to calculate everything as fast as possible – 30 s is very short when there is a timer in your face!”
	Moving from formulas to gestalt estimates was different	“I am used to calculating PPV, NPV, so it felt weird learning to intuit it”
Has GBL changed the way you think of diagnosis? (36/60 positive)	GBL emphasized the role of medical decision making for diagnosis	“It made me realize how important assessing the patient thoroughly and using the entire presentation of the patient to take into account the potential diagnosis vs. a particular aspect greatly impacts how to interpret tests,”
	Avoid unnecessary testing	Instead of just ordering something directly just because I have a suspicion of something, it’s also important to consider the probabilities behind this and avoid unnecessary testing”
	Understand patterns around use of tests	“This helped me pick up patterns quicker to understand what it means when a test is a certain % sensitive or specific. This will help me think quicker and on the fly in the future”
Do you believe you will practice any differently when performing diagnostic testing based on this training? (38/60 positive)	Will consider pretest probability when making a diagnosis	“Will be factoring in pre-test probabilities more often and whether a positive test would change my management”
	Will order tests more carefully	“It is a reminder to think more critically about the tests I order and how they will actually help develop a diagnosis”
	Will change how tests are interpreted	“I will use testing as a tool to aid in diagnoses, as opposed to jumping straight to conclusions based on results”

GBL, game based learning.

## Discussion

In this pilot study, an online GBL intervention nearly doubled diagnostic accuracy in medical trainees and practicing clinicians and persisted for three months. Follow up emails with cases did not improve accuracy. Participants found the training generally easy, enjoyable, and reported that GBL improves diagnostic test interpretation.

The primary goal of this pilot study was to determine the effect size and optimal intervention with GBL. We found an unexpectedly large effect size on a case-based score of diagnostic accuracy. GBL led to a near doubling of diagnostic accuracy. The effect of GBL persisted at one and three months without additional training. This large effect size means that in future RCTs, sample sizes required to study this accuracy outcome will be relatively small raising the possibility of more clinically focused outcomes, such as clinician practice patterns. Follow up emails did not improve performance, likely, as noted by one participant because of ignoring emails. The large effect size also suggests that this online, experiential approach to teaching diagnosis by estimation with natural frequencies [10], [11], [12, 19] outperforms traditional calculations and should be considered for medical education.

Most participants reported GBL was enjoyable, improved test interpretation and that they would recommend it to a colleague. Estimating the probability of a diagnosis after testing uses Bayesian updating, a skill that is developed with repetitive practice. Achieving adequate practice on a game, requires that the game be acceptable and moderately entertaining [20, 21]. Some trainees expressed opinions that GBL would have been helpful for USMLE board review. The goal the game, to require gestalt estimates in a timeframe similar to that encountered in patient care was experienced as stressful for some participants.

### Limitations

Limitations to this pilot study include that the sample size was moderate and within a single medical system and may not be generalizable. The outcome scale of diagnostic accuracy has not been validated. Although GBL greatly improved mean accuracy score, there was variability in clinician scores in all groups. We do not know how lack of follow-up may have impacted results at one and three months. Finally, participants were reimbursed for completion of the study and attention may not be replicated with standard voluntary participation.

## Conclusions

Game-based learning is a promising intervention to improve diagnosis that was well received and is easily disseminated to medical training programs.

## Supplementary Material

Supplementary Material
