# Supplementary material for: Game-based learning to improve diagnostic accuracy: a pilot randomized-controlled trial
Source: Diagnosis (Berl). Author manuscript; Available in PMC 2024 May 8. (PMC11075046; doi:10.1515/dx-2023-0133)
Supplement: supplementary material [file NIHMS1980684-supplement-supplementary_material.docx]

**DIAGNOSTIC CASES**

(exact text of cases provided. Correct answer highlighted.)

1. An 80 yo man in the emergency room is being considered for cardiac ischemia. He has known coronary artery disease, diabetes and a known abnormal stress test. You estimate he has a 50% pretest risk for ischemia. He has a **negative** exercise stress echo (sensitivity 80%, specificity 85%).
**Approximately how likely is he to have cardiac ischemia?**

- <1%
- 1-4%
- 5-14%
- 15-29%
- 30-49%
- 50-69%
- 70-84%
- 85-100%

2. A 75 yo woman with coronary artery disease and past heart failure presents with breathlessness when lying flat on her back and leg swelling. You estimate she has approximately a 70% pretest chance of heart failure. She undergoes a BNP test (sensitivity 90%, specificity 75%) which is **negative** at 80.
**Approximately how likely is she to have heart failure?**

- <1%
- 1-4%
- 5-14%
- 15-29%
- 30-49%
- 50-69%
- 70-84%
- 85-100%

3. A 35 yo man presents with cough, fever and shortness of breath. You estimate he has a 30% pretest chance of pneumonia based on this history and physical exam. He has a chest X-ray (sensitivity ~80%, specificity 70%) that is **negative** for pneumonia.
**Approximately how likely is he to have pneumonia?**

- <1%
- 1-4%
- 5-14%
- 15-29%
- 30-49%
- 50-69%
- 70-84%
- 85-100%

4. A 60 yo man with heart transplant develops bilateral pneumonia. Invasive aspergillosis is being considered. You estimate he has a 20% pretest chance of invasive aspergillosis. He has a **negative** serum galactomannan test (sensitivity 80%, specificity ~80%).
**Approximately how likely is he to have invasive aspergillosis?**

- <1%
- 1-4%
- 5-14%
- 15-29%
- 30-49%
- 50-69%
- 70-84%
- 85-100%

5. A 19 yo college student without signs or symptoms of genitourinary disease has undergone “STD screening”. You estimate she has a 0.1% pretest chance of syphilis. She has a **positive** syphilis FTA-ABS test (sensitivity 96%, specificity 95%).
**Approximately how likely is she to have syphilis?**

- <1%
- 1-4%
- 5-14%
- 15-29%
- 30-49%
- 50-69%
- 70-84%
- 85-100%

6. A 43 yo woman with intermittent, mild chest pain unrelated to activity and no risk factors. You estimate she has a 1% chance of ischemia. She has a **positive**HS troponin test (sensitivity 99%, specificity ~90%).

**How likely is she to have ischemia as the cause of her chest pain?**

- <1%
- 1-4%
- 5-14%
- 15-29%
- 30-49%
- 50-69%
- 70-84%
- 85-100%

7. A 50 yo woman with macular rash and joint pain is being considered for systemic lupus erythematosus (SLE). You estimate she has a 5% pretest chance of SLE. She has a **positive** ANA (>1:160) test (sensitivity 96%, specificity 86%).
**Approximately how likely is she to have SLE?**

- <1%
- 1-4%
- 5-14%
- 15-29%
- 30-49%
- 50-69%
- 70-84%
- 85-100%

8. A 50 yo man is being evaluated for COVID-19. His partner at home was recently diagnosed with COVID-19 and he has loss of taste and fatigue. You estimate he has a 80% pretest chance of COVID-19. He has a **negative** SARS-COV2 antigen test (sensitivity 60%, specificity 98%). 

**Approximately how likely is he to have COVID-19?**

- <1%
- 1-4%
- 5-14%
- 15-29%
- 30-49%
- 50-69%
- 70-84%
- 85-100%

9. A 55 yo man in the hospital develops mild diarrhea. You estimate he has a 1% pretest chance of C. difficile infection. He has a **positive** C. difficile PCR (sensitivity 99%, specificity ~90%).
 **Approximately how likely is he to have *C. difficile* infection?**

- <1%
- 1-4%
- 5-14%
- 15-29%
- 30-49%
- 50-69%
- 70-84%
- 85-100%

10. A 60 yo woman with fatigue is seen in the clinic. She is being considered for hypothyroidism. You estimate she has a 2% chance of hypothyroidism. She has a **positive** test for hypothyroidism, an elevated TSH (sensitivity 98%, specificity 92%).
**Approximately how likely is she to have hypothyroidism?**

- <1%
- 1-4%
- 5-14%
- 15-29%
- 30-49%
- 50-69%
- 70-84%
- 85-100%
